# Dataset of the application of handheld NIR and machine learning for chicken fillet authenticity study

**DOI:** 10.1016/j.dib.2020.105357

**Published:** 2020-02-29

**Authors:** Geert van Kollenburg, Yannick Weesepoel, Hadi Parastar, André van den Doel, Lutgarde Buydens, Jeroen Jansen

**Affiliations:** a Department of Chemistry, Sharif University of Technology, Tehran, Iran; b Radboud University, Department of Analytical Chemistry/ Chemometrics, Institute for Molecules and Materials (IMM), Heyendaalseweg 135, 6525 AJ Nijmegen, the Netherlands; c Wageningen Food Safety Research (WFSR), Wageningen University & Research, Wageningen, the Netherlands

**Keywords:** Handheld near-infrared, Chicken breast fillet, Meat authenticity, Growth conditions, Chemometrics, Ensemble learning

## Abstract

Diffuse reflectance near-infrared (NIR) data (908–1676 nm) of chicken breast fillets was recorded in a non-destructive way using a portable miniaturised NIR spectrometer. The NIR data was used to discriminate between fresh and thawed breast fillets and to determine the birds’ growth conditions. NIR data was recorded of 153 commercial supermarket chicken fillet samples by applying the NIR device equipped with the standard issue collar on the samples in three different ways: (i) directly on the meat (ii) through the top foil of the package (i.e. with an air pocket between the foil and the breast fillet), and (iii) through the top foil with the packaging turned bottom up (i.e. no air pocket between the foil and the breast fillet). In order to generate thawed samples, the fresh samples were frozen and subsequently thawed. The freshness of the fillets was checked using β-hydroxyacyl-CoA-dehydrogenase of 13% of the sample set. Five NIR spectra were collected per measurement mode from each sample resulting in 4590 raw NIR spectra. Multivariate statistics was applied and the interpretation of these calculations can be found in Parastar et al. [1]. The NIR data has a reuse potential for follow-up studies of chicken breast fillet authentication using a similar brand NIR device or to serve as calibration transfer data.

**Table:** Specifications Table

Subject	Food Science, Food Control
Specific subject area	Portable Near-Infrared spectroscopy data of chicken breast fillets for freshness and growth system authentication
Type of data	Table
How data were acquired	Portable Near-Infrared spectroscopy. MicroNIR Pro NIR (Viavi Solutions, Milpitas, CA, USA), powered by MicroNIR Pro software (version 2.2, Viavi Solutions) in diffuse reflectance mode in wavelength range of approximately 908–1676 nm with an evenly distributed spectral resolution, resulting in 125 variables/measurement
Data format	Raw
Parameters for data collection	A 99% white diffuse reflectance standard was used for calibration followed by a dark measurement. This calibration was repeated in 10 minute cycles. Samples were at a temperature of approximately +4 °C.
Description of data collection	NIR data was recorded by applying the NIR device equipped with the standard issue collar on the samples in three different ways: (i) directly on the meat, (ii) through the top foil of the package (i.e. with an air pocket between the foil and the breast fillet) and (iii) through the top foil with packaging turned bottom up (i.e. no air pocket between the foil and the breast fillet). Five replicates were recorded per sample, per measuremtent mode, measured on different locations on the fillets.
Data source location	Institution: Wageningen Food Safety Research City: Wageningen Country: The Netherlands Latitude and longitude GPS 51.9822598,5.6565744
Data accessibility	Public repository Repository name: Mendeley Data DOI: https://doi.org/10.17632/cp2hdhkys3 [2]
Related research article	Parastar et al., Integration of handheld NIR and machine learning for the development of a “Measure & Monitor” technology for chicken meat authenticity, Food Control 112 (2020). DOI: https://doi.org/10.1016/j.foodcont.2020.107149

**Table:** 

**Value of the Data** • NIR spectral databases require high amount of unique samples covering the analytical range and natural variability within the products (i.e. season, animal breed, growing system, animal slaughter age, etc.) in order to ensure that unknown samples are predicted correctly. A common bottleneck in NIR studies are that sample sets only meet minimum requirements [3]. Availability of NIR datasets from other studies using similar equipment and similar food products are therefore of importance for usage as external validation data or to supplement the NIR data-set of the new study. • The behaviour or reproducibility of two similar NIR instruments (when the instruments settings are similar) can be investigated. • This data can be used in calibration transfer studies from the NIR instrument used in this study to a different NIR instrument. • Scientists who work with NIR, especially the NIR equipment used in combination with food control and meat quality inspection and are in need of data of certified chicken breast fillets. The current data can be an addition or a source of external validation data for the chemometric models established. • NIR spectral databases are known to be specific for a certain NIR instrument coupled to a specific product. Studies concerning the improving this limitation might be in need of such data and can take advantage in using this data for training, validation or establishment of calibration transfer protocols.

## 1 Data description

This dataset contains data that were collected through Near-Infrared spectroscopy of chicken breast fillets and consists of the file: Data-Parastar et al., 2020 Food Control.csv which is a single spreadsheet of near-infrared spectra coupled to a number of discrete reference values [2].

Explanation of variables.

Sample_number: Sample number (as given by analyst).

Production_sytem: Production system of chicken breast fillet as indicated by the producer.

# CONV = Conventional chicken.

# 1 star = Free-range.

# 2 stars = Specialty.

# ORG = Organic, 3 stars.

# STD = Standard chicken.

# FR = Free range.

# CF = Corn fed chicken.

# MAR = Marinated chicken.

Scan_type: method of acquiring near-infrared spectrum.

# OM = On meat.

# TP = Through package.

# TB = Through package turned bottom up.

Freshness: Freshness of the chicken breast fillet.

# FR = Fresh.

# TH = Thawed.

X908.1/ … /X1676.2: Near-infrared variables.

## 2 Experimental design, materials, and methods

From Parastar et al., 2020 [1]:

“Samples (153 total) were shipped in ‘fresh packs’, guaranteeing a temperature between 4 and 7 °C for 96h. Samples arrived within that time-span. Marinated chicken fillets acted as controls, since these were expected to be highly identifiable” [1].

“NIR data was acquired using a MicroNIR Pro (Viavi Solutions, Milpitas, CA, USA), powered by the MicroNIR Pro software (version 2.2, Viavi Solutions) in diffuse reflectance mode in wavelength range of approximately 908–1676 nm with an evenly distributed spectral resolution, resulting in 125 variables/measurement.” [1].

“The 153 chicken fillet samples were subjected to non-destructive NIR measurements by applying the NIR with standard collar in three different ways: on meat (*OM*), through package (*TP*) and through packaging bottom up (*TB*).” [1].

“Five replicates were taken per *OM/TP/TB*, with a total of 4590 raw NIR measurements.” [1].

A more detailed description of the work, experimental design, materials, and methods can be found in Parastar et al., 2020 [1].
